# Feedback loop LINC00511–YTHDF2–SOX2 regulatory network drives cholangiocarcinoma progression and stemness

**DOI:** 10.1002/mco2.743

**Published:** 2024-10-22

**Authors:** Canghai Guan, Xinlei Zou, Xin Gao, Sidi Liu, Jianjun Gao, Wujiang Shi, Qingfu Dong, Xingming Jiang, Xiangyu Zhong

**Affiliations:** ^1^ Department of General Surgery The 2nd Affiliated Hospital of Harbin Medical University Harbin Heilongjiang Province China

**Keywords:** cholangiocarcinoma, LINC00511, long noncoding RNA, PDX, SOX2, tumorigenic stemness, YTHDF2

## Abstract

Cholangiocarcinoma (CCA) was identified as a malignant tumor with rising incidence and mortality rates, and the roles of long noncoding RNA (lncRNA) in CCA remained not entirely clear. In this study, LINC00511 had high expression in CCA, which was closely related to poor prognosis. Knockdown of LINC00511 significantly inhibited cell malignant biological behaviors. It also affected the stemness of CCA, evidenced by decreased SOX2 protein expression. Moreover, the study revealed the interaction of LINC00511, YTHDF2, and SOX2 in CCA. Specifically, LINC00511 facilitated the formation of a complex with YTHDF2 on SOX2 mRNA, which uniquely enhances the mRNA's stability through m6A methylation sites. This stabilization appears crucial for maintaining malignant behaviors in CCA cells. Additionally, LINC00511 modulated SOX2 expression via the PI3K/AKT signaling pathway. Meanwhile, SOX2 can also promote LINC00511 expression as an upstream transcription factor, thereby confirming a positive feedback loop formed by LINC00511, YTHDF2, and SOX2, which plays a significant role in the occurrence and development of CCA. Finally, the study successfully constructed two patient‐derived xenograft models, revealing the vital role of LINC00511 in CCA development. In summary, this research provides a comprehensive understanding of the role of LINC00511 in the pathogenesis of CCA.

## Graphical abstract

LINC00511 significantly infected cell proliferation, migration, invasion, and angiogenesis. The interaction of LINC00511, YTHDF2, and SOX2 in CCA. LINC00511 formed a complex with YTHDF2, affecting the stability of SOX2 mRNA and thus influencing SOX2 expression. LINC00511 further modulated the expression of SOX2 through the PI3K/AKT signaling pathway. Meanwhile, SOX2 can also promote LINC00511 expression as an upstream transcription factor, thereby confirming a positive feedback loop formed by LINC00511, YTHDF2, and SOX2, which plays a significant role in the occurrence and development of CCA.

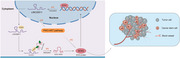

## 1 INTRODUCTION

Cholangiocarcinoma (CCA) was recognized as one of the most prevalent and lethal digestive system cancers worldwide.^1^, ^2^, ^3^ The bile duct, a critical component of the liver responsible for secreting and transporting bile, was the primary site of cholangiocarcinoma occurrence, particularly in the epithelial cells of the bile duct.^4^, ^5^, ^6^ The incidence of CCA was rising annually, posing a grave threat to patient health and survival rates.^7^, ^8^, ^9^ However, the inconspicuous early symptoms of CCA and the absence of efficacious early diagnosis methods meant that most patients were diagnosed at an advanced stage, which severely impacted survival outcomes.^10^, ^11^ The evolution and advancement of CCA involve a multifaceted interplay of biological processes. This intricate orchestration encompasses diverse mechanisms, including epigenetic modifications, signaling pathway dysregulation, and immune microenvironment modulation.^6^, ^12^ These processes jointly contributed to the genesis and progression of CCA, emphasizing the complexity of its pathogenesis. Thus, a thorough exploration of the pathogenesis of CCA had significant implications for improving patient outcomes.

Long noncoding RNA (lncRNA), defined as RNA sequences exceeding 200 nucleotides in length without encoding proteins, was proven to have a substantial impact on gene regulation.^13^ It was known to modulate gene expression by interacting with other molecules, such as DNA, RNA, and proteins.^14^, ^15^ Recent research has underscored the significant roles of lncRNAs in various biological processes such as cell proliferation, migration, apoptosis, and differentiation.^16^, ^17^, ^18^ Furthermore, lncRNAs played pivotal roles in the onset, development, and metastasis of tumors.^19^, ^20^, ^21^

LINC00511, a recognized lncRNA, was found to be abnormal expressed in diverse cancers, including breast, lung, and gastric cancer.^22^ For example, in breast cancer, LINC00511 was shown to foster progression by competitively binding miR‐150 with MMP13^23^; in colorectal cancer, LINC00511 acted as a carcinogen by suppressing IL‐24 expression through interaction with EZH2^24^; additionally, LINC00511 facilitated carcinogenesis by recruiting EZH2 to the PTEN promoter, enhancing methylation of the PTEN promoter, and activating downstream signaling pathways influencing gastric cancer proliferation.^25^ However, studies on the role and mechanism of LINC00511 in CCA remain scarce. Thus, our research sought to investigate the expression of LINC00511 and its potential biological functions and mechanisms.

Our investigation revealed that LINC00511 was upregulated in CCA, and its expression level was closely linked to the clinical characteristics and prognosis of patients. We further determined that LINC00511 could modulate the proliferation, migration, invasion, and tumor stemness of tumor cells by interacting with YTHDF2 and SOX2, thus impacting SOX2 mRNA stability through m6A methylation‐a novel finding that adds a critical layer to our understanding of its regulatory mechanisms. Concurrently, we ascertained that LINC00511 could alter the expression of SOX2 through the PI3K/AKT signaling pathway. Moreover, SOX2, acting as the upstream transcription factor of LINC00511, influenced the malignant biological behaviors of tumors. These findings illuminated the significant role of LINC00511 in the onset and progression of CCA, providing valuable insights for subsequent research on the role and mechanism of LINC00511.

## 2 RESULTS

### 2.1 LINC00511 was directly associated with poor prognosis in cholangiocarcinoma

**FIGURE 1:**
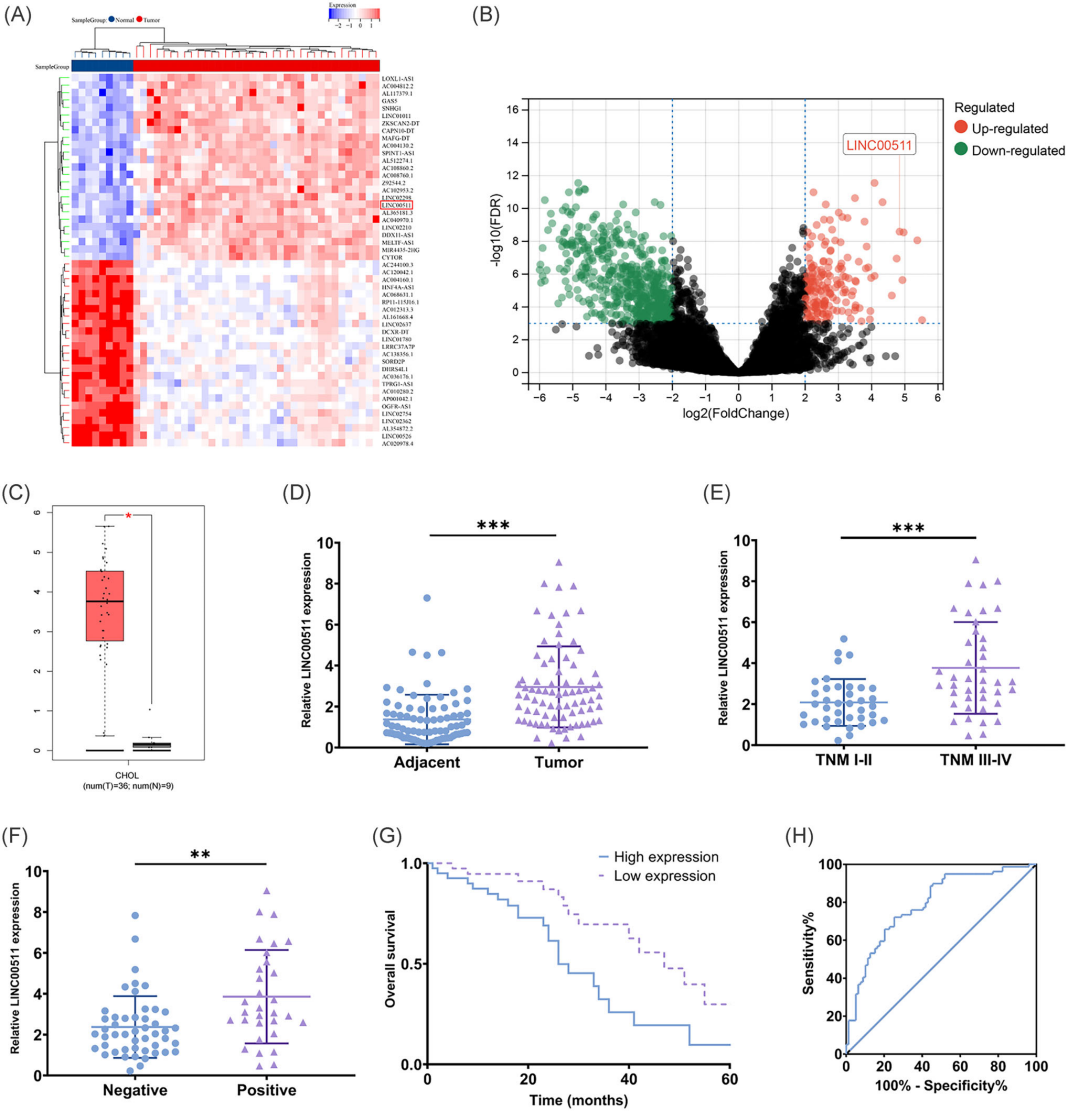
Expression patterns and clinical pathological characteristics of LINC00511 in CCA. (A, B) Heatmaps and volcano plots of high and low expression lncRNA from the TCGA database. (C) Expression level of LINC00511 in CCA derived from the GEPIA website. (D) Relative expression of LINC00511 in CCA tissues and adjacent tissues (*n *= 79). (E) Correlation between LINC00511 relative expression and patients with different TNM stages. (F) Association between LINC00511 expression and patients with lymph node metastasis. (G) The overall survival of patients with high and low expression of LINC00511. (H) Utilizing the ROC curve to evaluate the prognostic potential of LINC00511 for CCA patients (AUC = 0.7906). **p < 0.01, ***p < 0.001. CCA, cholangiocarcinoma.

We initially identified a significant increase in the expression of LINC00511 in CCA through a comprehensive bioinformatic analysis of the TCGA database (Figure 1A–C). To confirm this finding, we further assessed the expression of LINC00511 in tissue samples from our specimen repository, and the results confirmed a notable increase in LINC00511 expression in CCA (Figure 1D). Furthermore, we discovered that high expression of LINC00511 was strongly correlated with TNM stage (Figure 1E) and lymph node invasion (Figure 1F), with those exhibiting high LINC00511 expression having a poorer prognosis (Figure 1G, H). To explore further the impact of LINC00511 overexpression on the prognosis of CCA patients, we segregated the patients into high‐expression and low‐expression groups based on the expression levels of LINC00511. We conducted an examination of clinical pathological features as well as univariate and multivariate analyses. The results demonstrated a strong association between the high TNM stage, lymph node invasion, and high LINC00511 expression (Table S2). Additionally, lymph node invasion and the abnormal upregulation of LINC00511 were identified as independent risk factors for an adverse prognosis in patients (Table S3).

### 2.2 Critical role of LINC00511 in tumor growth

**FIGURE 2:**
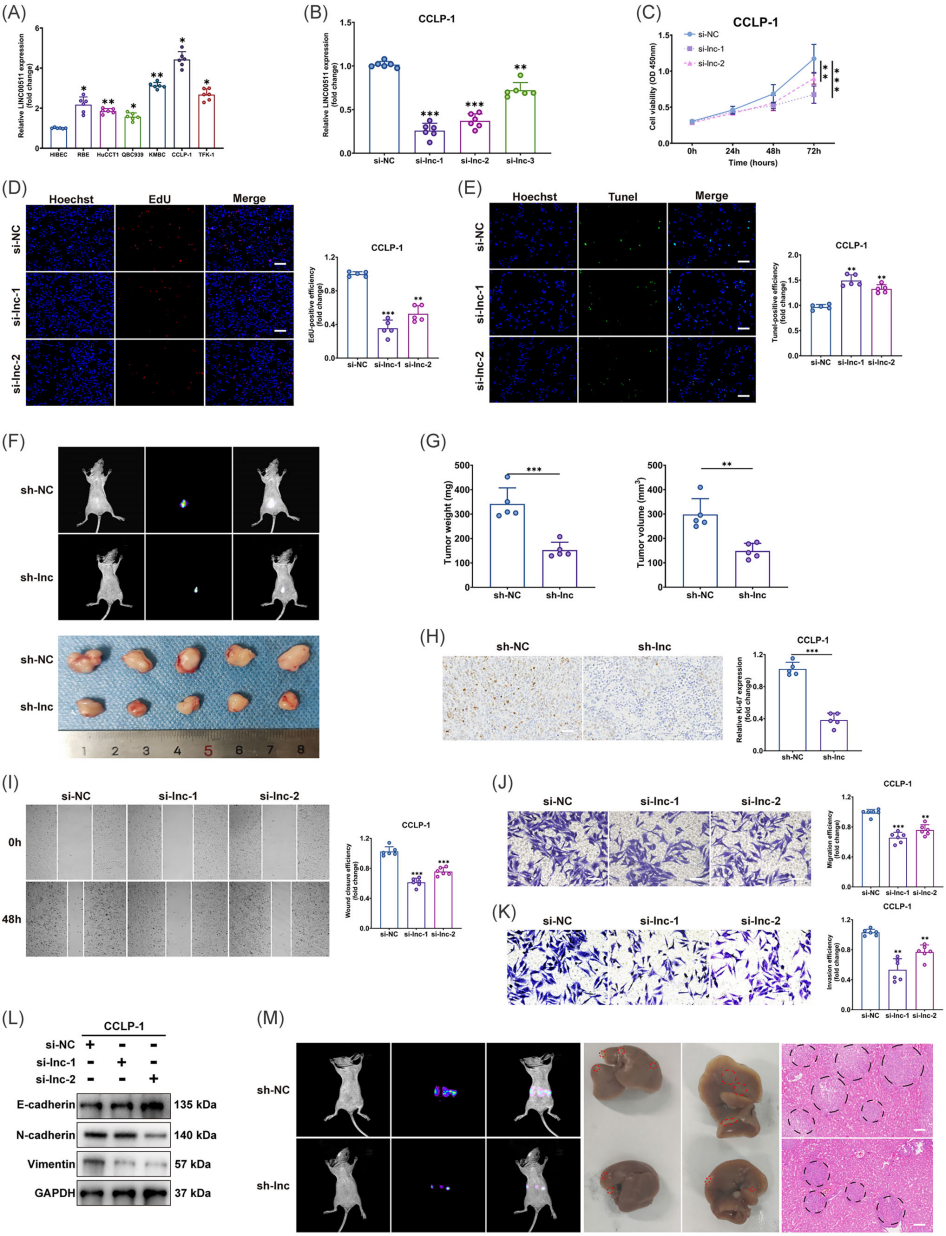
Impact of LINC00511 on CCA proliferation, apoptosis, migration, and invasion. (A) Expression of LINC00511 in CCA cell lines (*n *= 6). B Intervention efficiency after transfection with three LINC00511 siRNAs (*n *= 6). (C, D) Assessment of proliferation alterations in CCLP‐1 through CCK‐8 and EdU assays (*n *= 6). (E) Apoptosis alterations of CCLP‐1 evaluated via TUNEL assays (*n *= 6). (F) Representative images of subcutaneous tumors in nude mice post LINC00511 silencing. (G) Weight and volume of subcutaneous tumors after silencing LINC00511 (*n *= 5). (H) Subcutaneous tumor tissues from different groups were subjected to Ki‐67 staining (*n *= 5). (I, J) Wound healing and transwell migration assays gauging the migratory capability of CCLP‐1 (*n *= 6). (K) Transwell invasion assays determining the invasive potential of CCLP‐1 (*n *= 6). (L) Western blot analysis of epithelial–mesenchymal transition‐related markers. (M) Representative images of liver metastasis following silencing of LINC00511. *p < 0.05, **p < 0.01, ***p < 0.001. CCA, cholangiocarcinoma.

In our investigation, we initially measured the expression of LINC00511 in six types of CCA cells. Our analyses showed that, in comparison to normal bile duct cells, LINC00511 expression was markedly elevated in all examined tumor cells (Figure 2A). To delve deeper into the function of LINC00511, we selected CCLP‐1, which exhibited the highest LINC00511 expression, for knockdown experiments (Figure 2B). Conversely, QBC939, which had the lowest LINC00511 expression, was used for overexpression transfection experiments (Figure S1A). We utilized CCK‐8 and EdU assays to evaluate cell proliferation capabilities. The results demonstrated that both LINC00511 knockdown and overexpression markedly altered the cells' proliferation capacity (Figure 2C, D; Figure S1B, C). The TUNEL assay further established that LINC00511 knockdown considerably enhanced apoptosis in CCLP‐1 cells (Figure 2E; Figure S1D). To further validate the in vivo function of LINC00511, we subcutaneous tumors were generated in nude mice using transfected CCLP‐1 cells (Figure 2F). Comparative analysis indicated that, relative to the control group, the weight and volume of the subcutaneous tumors in mice were significantly diminished following LINC00511 knockdown (Figure 2G). Conversely, the overexpression of LINC00511 exhibited opposite effects (Figure S1E, F). Moreover, these findings were supported by subsequent Ki‐67 staining results, which confirmed increased proliferation in the overexpression group and decreased proliferation in the knockdown group (Figure 2H; Figure S1G).

### 2.3 LINC00511's role in enhancing tumor metastatic behavior

The effects of LINC00511 on the migratory capabilities of CCA cells were examined through wound‐healing and transwell migration assays. Experimental results indicated that the migration potential of tumor cells was significantly reduced following LINC00511 knockdown (Figure 2I, J), whereas LINC00511 overexpression notably enhanced this capacity (Figure S2A, B). We further assessment of LINC00511's role in cell invasiveness using transwell invasion assays. These findings confirmed that LINC00511 knockdown led to a substantial decrease in the invasive capabilities of CCLP‐1 cells (Figure 2K), while its overexpression produced the opposite effect (Figure S2C). Additionally, the process of epithelial–mesenchymal transition, where epithelial cells acquire mesenchymal traits that enhance their migratory and invasive abilities,^26^, ^27^ was significantly influenced by LINC00511 according to our experiments (Figure 2L; Figure S2D). To further explore this in a physiological context, we implemented in vivo hepatic metastasis imaging and hematoxylin and eosin (H&E) staining techniques to assess the role of LINC00511 in promoting tumor metastasis. Results demonstrated that LINC00511 knockdown markedly curtailed hepatic metastasis (Figure 2M), while its overexpression amplified these effects (Figure S2E). Collectively, these results underscore LINC00511's critical function in driving tumor cell migration, invasion, and subsequent metastasis.

### 2.4 The impact of LINC00511 on tumor stemness and angiogenesis

**FIGURE 3:**
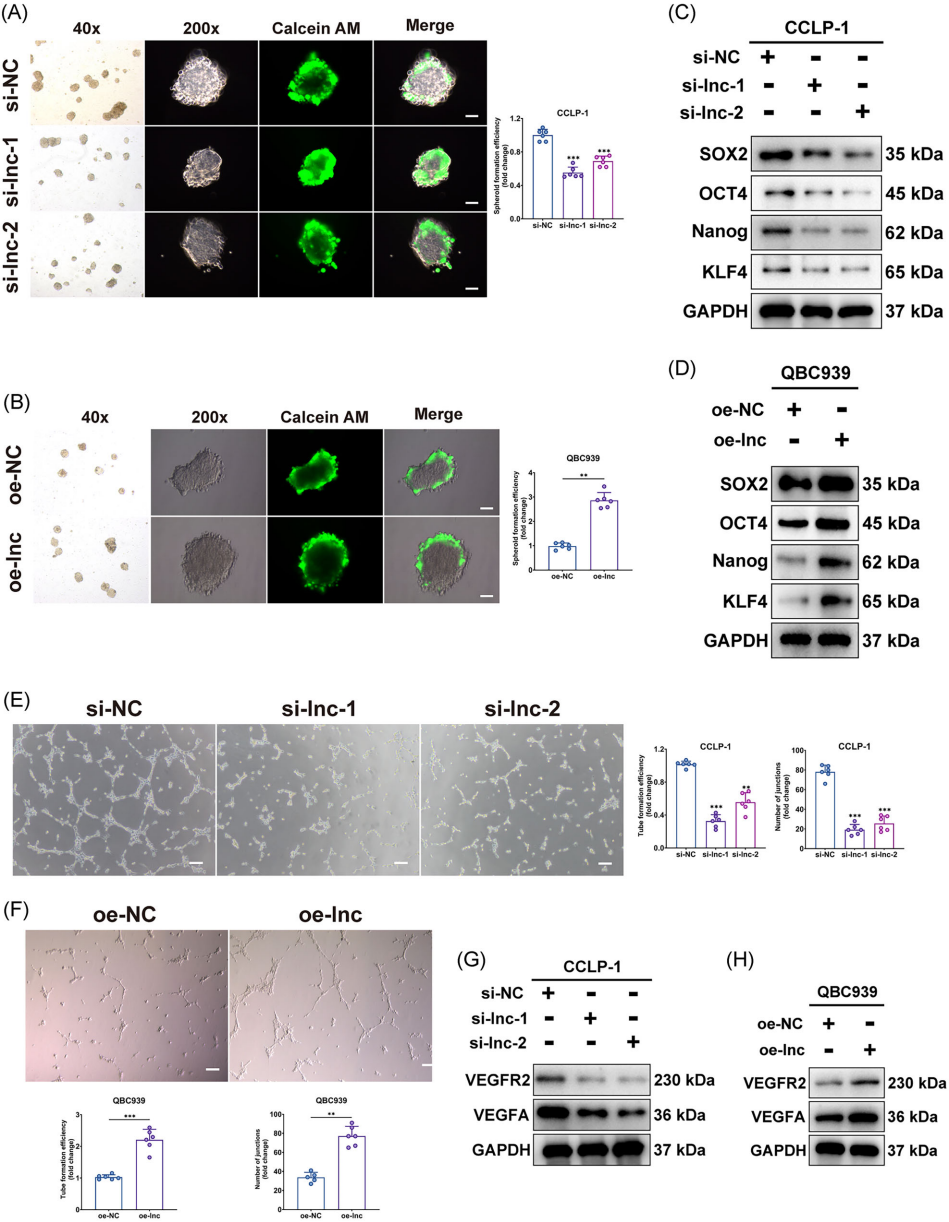
Effects of LINC00511 on CCA tumor stemness and angiogenesis. (A, B) Tumor sphere formation assays evaluating the sphere‐forming efficiency of CCA cells (*n *= 6). (C, D) Western blot analysis focusing on alterations in the expression of stem cell‐related markers. (E, F) Angiogenesis assays assessing the impact of silencing and overexpressing LINC00511 on CCA conditioned medium's effect on endothelial cell formation (*n *= 6). (G, H) Western blot analysis focusing on alterations in the expression of markers of angiogenesis. *p < 0.05, **p < 0.01, ***p < 0.001. CCA, cholangiocarcinoma.

The tumor sphere formation assay, used to evaluate the stemness of tumor cells, was employed to examine the self‐renewal and multidirectional differentiation capabilities of tumor cells. These capabilities were integral to the development and metastasis of tumors.^28^, ^29^ Experimental results showed a significant influence of LINC00511 on the cell's sphere‐forming capacity. Specifically, knocking down LINC00511 reduced the sphere‐forming ability (Figure 3A) while overexpressing LINC00511 boosted it (Figure 3B). To further our understanding of how LINC00511 impacts CCA stemness, the protein expression of stemness‐associated markers such as SOX2, OCT4, Nanog, and KLF4 was analyzed (Figure 3C, D). These vital factors in maintaining tumor stemness were markedly decreased after knocking down LINC00511, reinforcing the role of LINC00511 in affecting stemness.

Additionally, the effect of LINC00511 on the capability of tumor cells to stimulate endothelial cell tube formation was assessed through an angiogenesis assay. Angiogenesis, essential for tumor growth and metastasis,^30^ was notably diminished after knocking down LINC00511 and amplified after overexpressing it (Figure 3E, F). In addition, knockdown or overexpression of LINC00511 inhibited and promoted the expression of important molecules involved in angiogenesis, respectively (Figure 3G, H). These results indicated that LINC00511 might also play a role in tumor development by impacting the stemness and the capability of CCA cells to promote endothelial cell tube formation.

### 2.5 The interactions of LINC00511, YTHDF2, and SOX2

**FIGURE 4:**
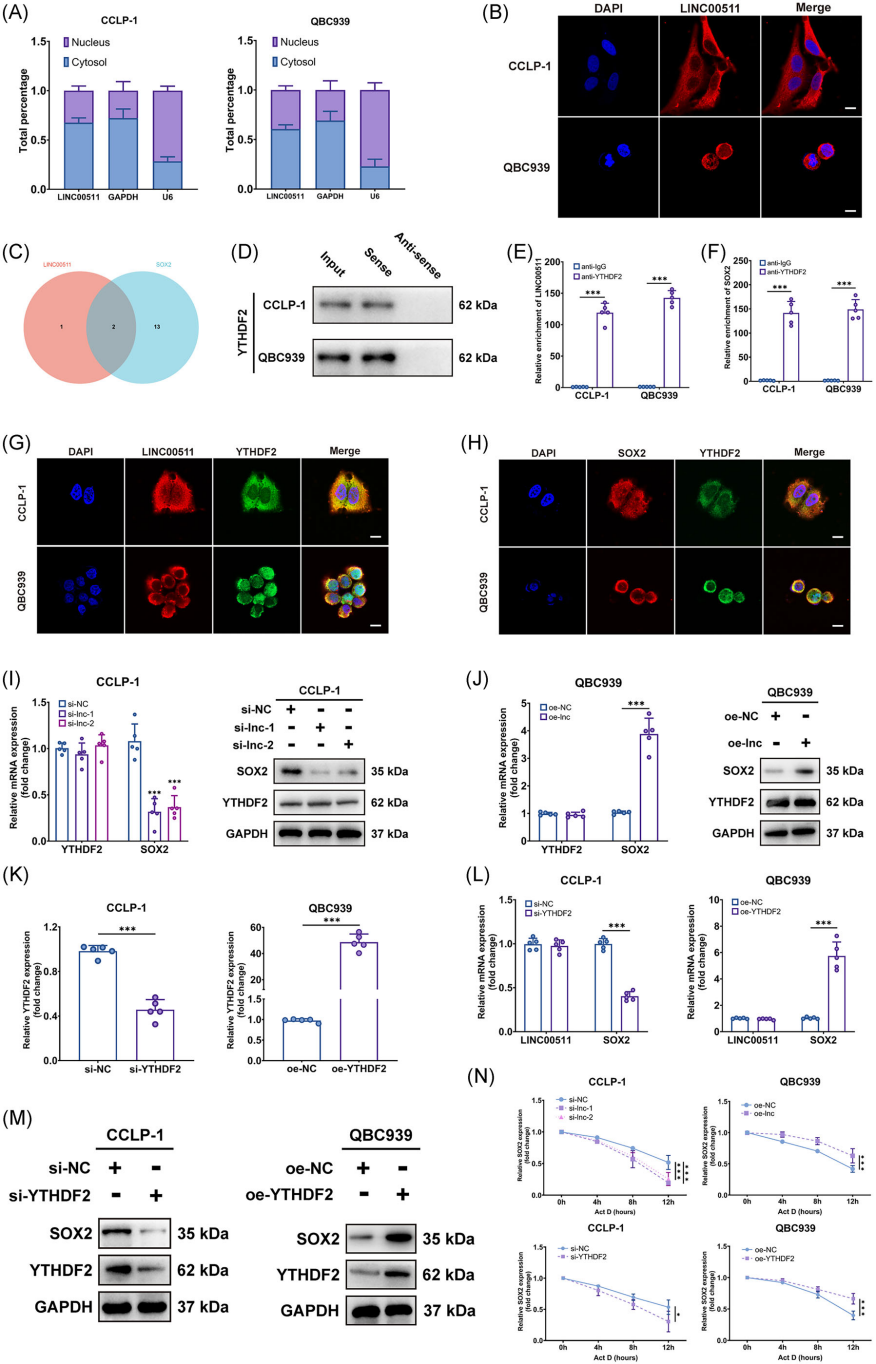
The interactions of LINC00511, YTHDF2, and SOX2. (A, B) Subcellular localization and FISH assays identified the localization of LINC00511 in CCLP‐1 and QBC939 (*n *= 3). (C) StarBase predicted potential binding proteins between LINC00511 and SOX2. (D) RNA pull‐down assays verified the binding of LINC00511 to YTHDF2. (E, F) RIP assay confirmed the enrichment of YTHDF2 with SOX2 and LINC00511 (*n *= 5). (G, H) FISH and IF showed the co‐localization of LINC00511, YTHDF2 protein and SOX2 mRNA in cholangiocarcinoma cells. (I) YTHDF2 and SOX2 mRNA and protein expression change post LINC00511 silencing (*n *= 5). (J) The mRNA and protein expression of YTHDF2 and SOX2 were analyzed after LINC00511 overexpression (*n *= 5). (K) qRT‐PCR ascertained the intervention transfection efficiency of YTHDF2 (*n *= 5). (L) Assessed LINC00511 and SOX2 mRNA expression change following YTHDF2 silencing and overexpression (*n *= 5). (M) Western blot detailed protein expression of YTHDF2 and SOX2 after YTHDF2 silencing and overexpression. (N) qRT‐PCR investigated the degradation rate of SOX2 mRNA following actinomycin D treatment (*n *= 5). *p < 0.05, ***p < 0.001. FISH, fluorescence in situ hybridization.

We further delved into the mechanism of action of LINC00511 in CCA. Initially, through subcellular localization and fluorescence in situ hybridization (FISH) assays, LINC00511 was predominantly identified in the cytoplasm (Figure 4A, B). This observation provides critical clues for further exploring the function of LINC00511, as lncRNAs in the cytoplasm often regulate gene expression by interacting with other molecules. Our study particularly focused on how LINC00511 affects tumor stemness, which is deeply intertwined with the occurrence, development, and metastasis of tumors. Earlier findings showed that LINC00511 significantly regulated the expression level of SOX2, a key protein of dryness (Figure 3C, D). Although the expressions of other dry markers such as OCT4, Nanog, and KLF4 were also affected by LINC00511, previous studies showed that SOX2 was the main upstream factor in the regulation of stemness.^31^ In addition, our supplementary experimental results showed that LINC00511 affected the mRNA expression level of SOX2, but did not affect the mRNA expression of OCT4, Nanog, KLF4 (Figure S3A).

LncRNAs usually impact the expression of downstream gene mRNAs through the competing endogenous RNA mechanism.^21^ However, predictions using the starBase database did not find intersecting microRNAs between LINC00511 and SOX2. This suggested that LINC00511 might affect the expression of SOX2 through other mechanisms. We then considered that RNA‐binding protein (RBP) might be involved in this process, as RBP could form complexes with RNA, thereby affecting the stability and translation efficiency of RNA. Predictions were made through the starBase database, and two proteins, YTHDF2 and ELAVL1, were found that might interact with both LINC00511 and SOX2 (Figure 4C). This prediction was verified through RNA pull‐down experiments and western blot experiments, and the results showed that only YTHDF2 could be detected (Figure 4D).

This is a significant discovery, as YTHDF2 was a key m6A reader that typically modulated mRNA stability through m6A modifications, and its binding could either destabilize certain transcripts or stabilize key oncogenes such as MYC and VEGF in an m6A‐dependent manner.^32^, ^33^, ^34^ Further investigation showed that the products pulled down by YTHDF2 antibodies exhibited a significant enrichment of LINC00511 and SOX2 when tested with qRT‐PCR (Figure 4E, F). Through confocal microscopy, we found that the localization of LINC00511 and YTHDF2 in cells was consistent (Figure 4G), mainly in the cytoplasm, and the location of the labeled with SOX2 mRNA and YTHDF2 protein fluorescence was also consistent (Figure 4H). These results confirmed the prediction that LINC00511, YTHDF2, and SOX2 might form a complex in CCA cells, thereby affecting the expression of SOX2. A subsequent study found that the mRNA and protein levels of SOX2 were significantly downregulated or upregulated by knocking down or overexpressing LINC00511, respectively, but the mRNA and protein expression of YTHDF2 was not affected (Figure 4I, J). Knockdown and overexpression of YTHDF2 led to downregulation and upregulation of SOX2 mRNA and protein levels in CCA cells, respectively, without affecting LINC00511 expression (Figure 4K–M). These results suggested that YTHDF2 might affect the expression of SOX2 by forming a complex with LINC00511. After treating cells with actinomycin D, it was found that silencing both YTHDF2 and LINC00511 accelerated the degradation of SOX2 mRNA while overexpressing both YTHDF2 and LINC00511 had the opposite effect (Figure 4N). This result suggested that LINC00511 and YTHDF2 regulate the expression of SOX2 by affecting the stability of SOX2 mRNA.

To further determine the role of m6A in YTHDF2 function, we used siRNA to knock out the m6A writers METTL3 and METTL14 (Figure S3B). Only the knockdown of METTL3 reduced SOX2 expression levels in CCA (Figure S3C), leading us to select METTL3 for methylated RNA immunoprecipitation (MeRIP)‐qPCR to measure the binding of YTHDF2 to SOX2 mRNA. As expected, knocking down METTL3 reduced m6A levels in CCA cells (Figure S3D) and also decreased the binding of YTHDF2 to SOX2 mRNA (Figure S3E). Further studies showed that knocking down YTHDF2 in normal bile duct cells (HIBEC) did not affect SOX2 mRNA levels but reduced them in CCLP‐1 cells (Figure S3F). A time‐course following treatment with actinomycin D showed that siRNA‐mediated knockdown of YTHDF2 specifically reduced the stability of SOX2 mRNA in CCLP‐1 cells with little effect on HIBEC (Figure S3G), thus indicating a CCA‐specific dependency where YTHDF2 supports cholangiocarcinoma cells.

### 2.6 The LINC00511 complex affected CCA malignant biological behaviors

**FIGURE 5:**
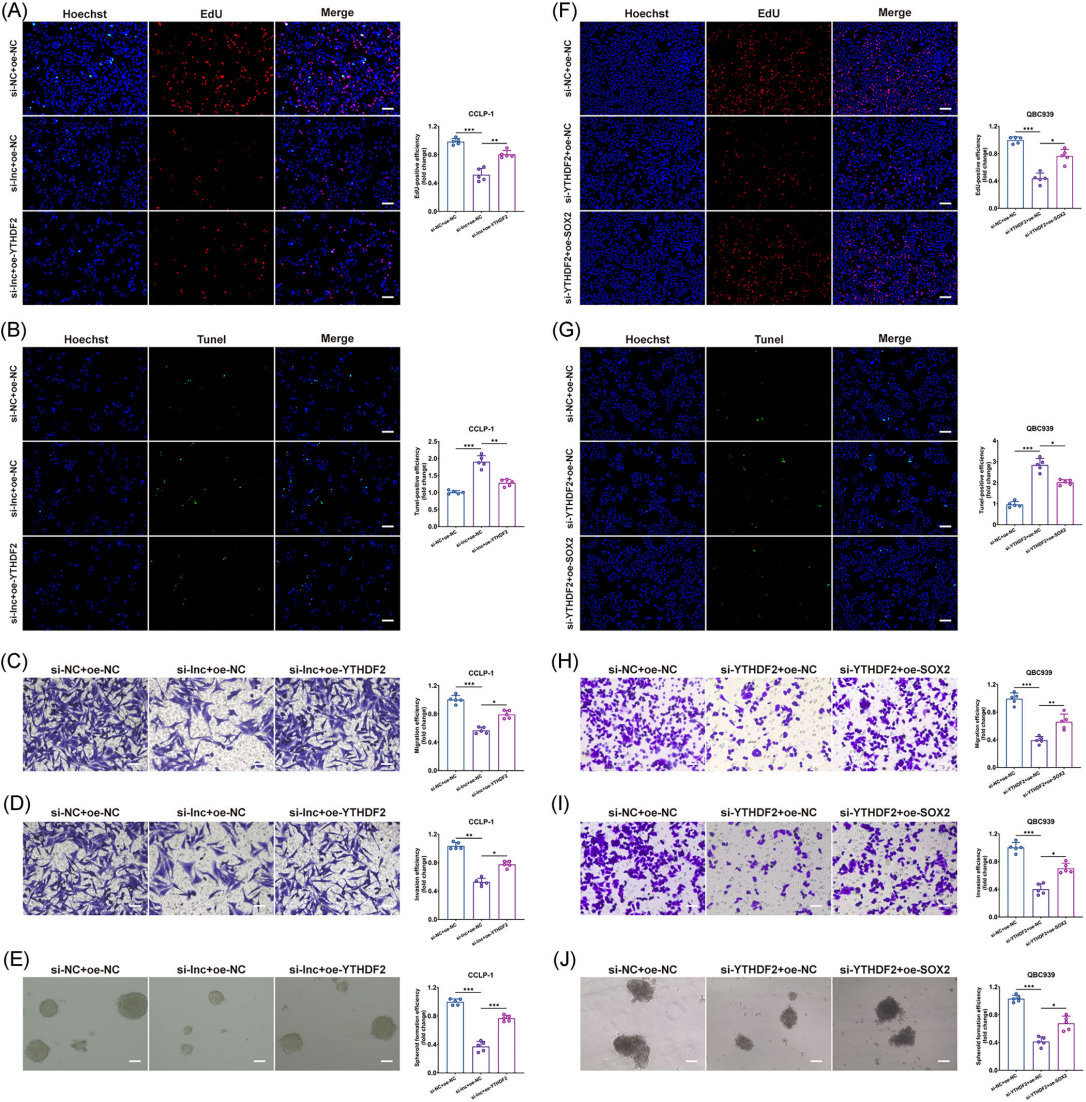
Regulatory effects of LINC00511, YTHDF2, and SOX2 on malignant biological behavior in cholangiocarcinoma. (A–E) The inhibitory effects of silencing LINC00511 on CCLP‐1 proliferation, anti‐apoptosis, migration, invasion, and stemness could be reversed by overexpressing YTHDF2 (*n *= 5). (F–J) The inhibitory effects of silencing YTHDF2 on QBC939 proliferation, anti‐apoptosis, migration, invasion, and stemness could be reversed by overexpressing SOX2 (*n *= 5). *p < 0.05, **p < 0.01, ***p < 0.001.

We continued to investigate how the interactions among LINC00511, YTHDF2, and SOX2 influenced the biological behaviors of CCA. In our experiments, we observed that the suppressive effects of LINC00511 on proliferation, anti‐apoptosis, migration, invasion, and stemness in CCLP‐1 cells could be counteracted by upregulated YTHDF2 (Figure 5A–E). Conversely, when YTHDF2 expression was reduced, the inhibitory impacts on proliferation, anti‐apoptosis, migration, invasion, and stemness in QBC939 cells could be reversed by the overexpression of SOX2, which in turn exacerbated the malignant biological behaviors of CCA (Figure 5F–J). These rescue experiments underscored SOX2 as a critical downstream effector molecule, potentially mediating the effects of YTHDF2 and LINC00511 on the malignant progression of tumors.

### 2.7 PI3K/AKT pathway regulated SOX2 in CCA

**FIGURE 6:**
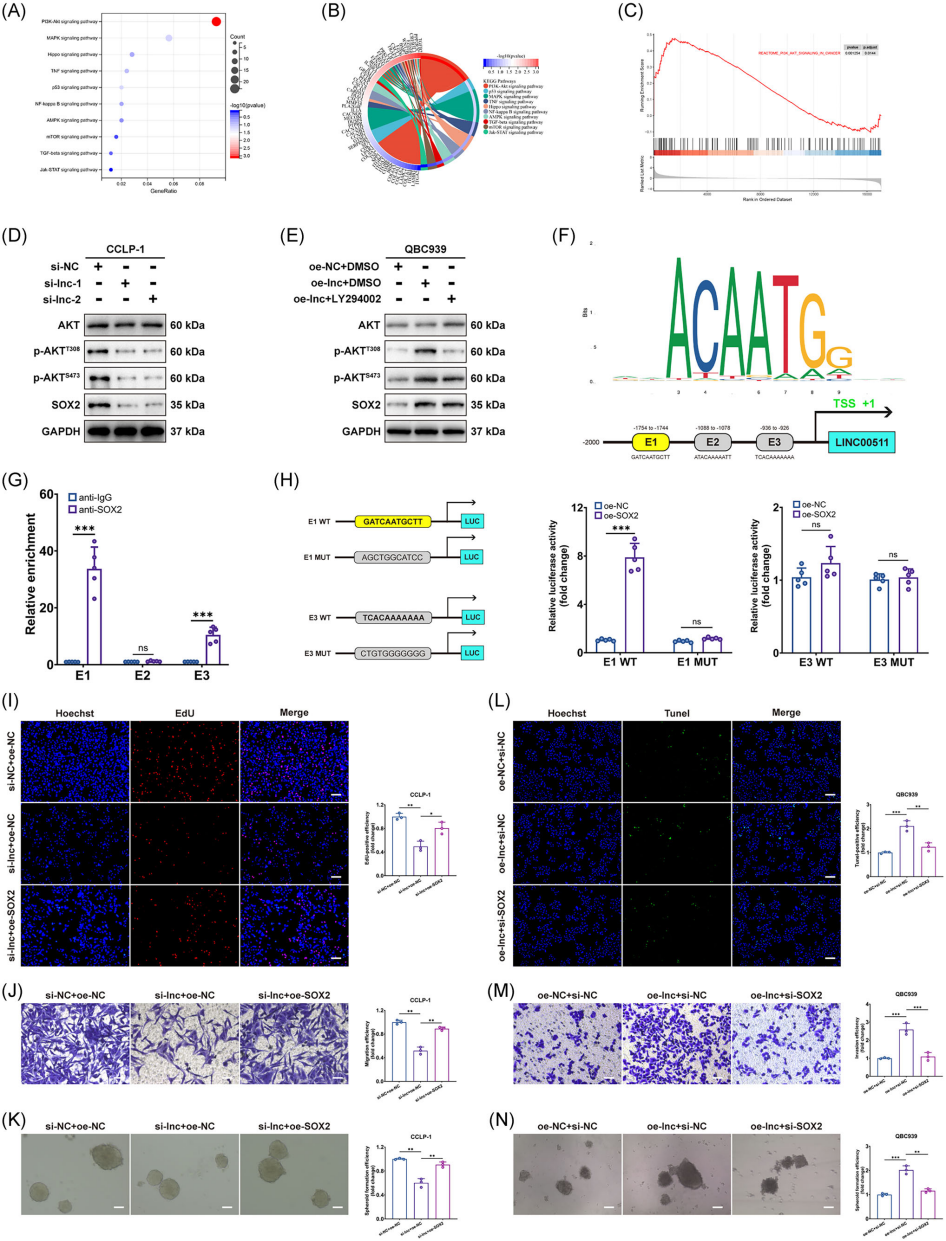
LINC00511 modulated SOX2 expression via the PI3K/AKT signaling pathway. (A–C) KEGG, GO, and GSEA analyses on LINC00511 grouping based on the TCGA database. (D) Western blot examined the expression of AKT, p‐AKT, and SOX2 post‐LINC00511 silencing. (E) Changes in protein expression following overexpression of LINC00511 and application of PI3K/AKT signaling inhibitors. (F) SOX2 motif and binding sites to LINC00511 promoter forecasted via JASPAR. (G) Chromatin immunoprecipitation (ChIP) assays measured SOX2 protein enrichment at respective binding sites (*n *= 5). (H) Luciferase reporter assay validated SOX2 binding with E1 and E3 (*n *= 5). (I–K) Inhibitory effects of LINC00511 silencing on CCLP‐1 proliferation, migration, and stemness were nullified by overexpressing SOX2 (*n *= 3). (L–N) The promotional effects of LINC00511 overexpression on QBC939 anti‐apoptosis, invasion, and stemness were undone by SOX2 silencing (*n *= 3). *p < 0.05, **p < 0.01, ***p < 0.001.

Bioinformatics analyses, including KEGG, GO, and GSEA, identified a link between LINC00511 and the PI3K/AKT signaling pathway (Figure 6A–C). This discovery was intriguing, as previous studies had confirmed that the PI3K/AKT pathway could promote SOX2 expression.^35^, ^36^ To explore the hypothesis that LINC00511 might influence SOX2 expression via the PI3K/AKT pathway in CCA, a knockdown of LINC00511 was performed. Observations showed that the expression of key proteins in the PI3K/AKT pathway and SOX2 was impacted (Figure 6D). Moreover, upon utilizing the widely used PI3K inhibitor LY294002, suppression of the upregulation of vital proteins in the PI3K/AKT pathway and SOX2 protein expression was observed (Figure 6E). Furthermore, SOX2 was identified as a transcription factor. Using the JASPER database, three potential binding sites (E1, E2, E3) for upstream promoter binding of SOX2 and LINC00511 were identified (Figure 6F). This finding was corroborated through chromatin immunoprecipitation (ChIP) experiments, with results showing notable enrichment at the E1 and E3 sites (Figure 6G). A subsequent luciferase reporter gene assay confirmed that E1 was the primary binding site (Figure 6H). In later experiments, the antitumor effect of silencing LINC00511 was reversed by overexpressing SOX2 (Figure 6I–K). Conversely, overexpressing LINC00511 promoted these effects, but silencing SOX2 reversed these outcomes (Figure 6L–N). Thus, our study revealed a novel regulatory mechanism, the LINC00511‐SOX2 positive feedback loop involving YTHDF2. This discovery not only provides new insights into the molecular mechanisms of CCA but also suggests potential targets for the development of innovative treatment strategies, such as drugs targeting LINC00511, YTHDF2, or SOX2 to disrupt this loop and curb tumor progression.

### 2.8 Targeted LINC00511 intervention via PDX model

**FIGURE 7:**
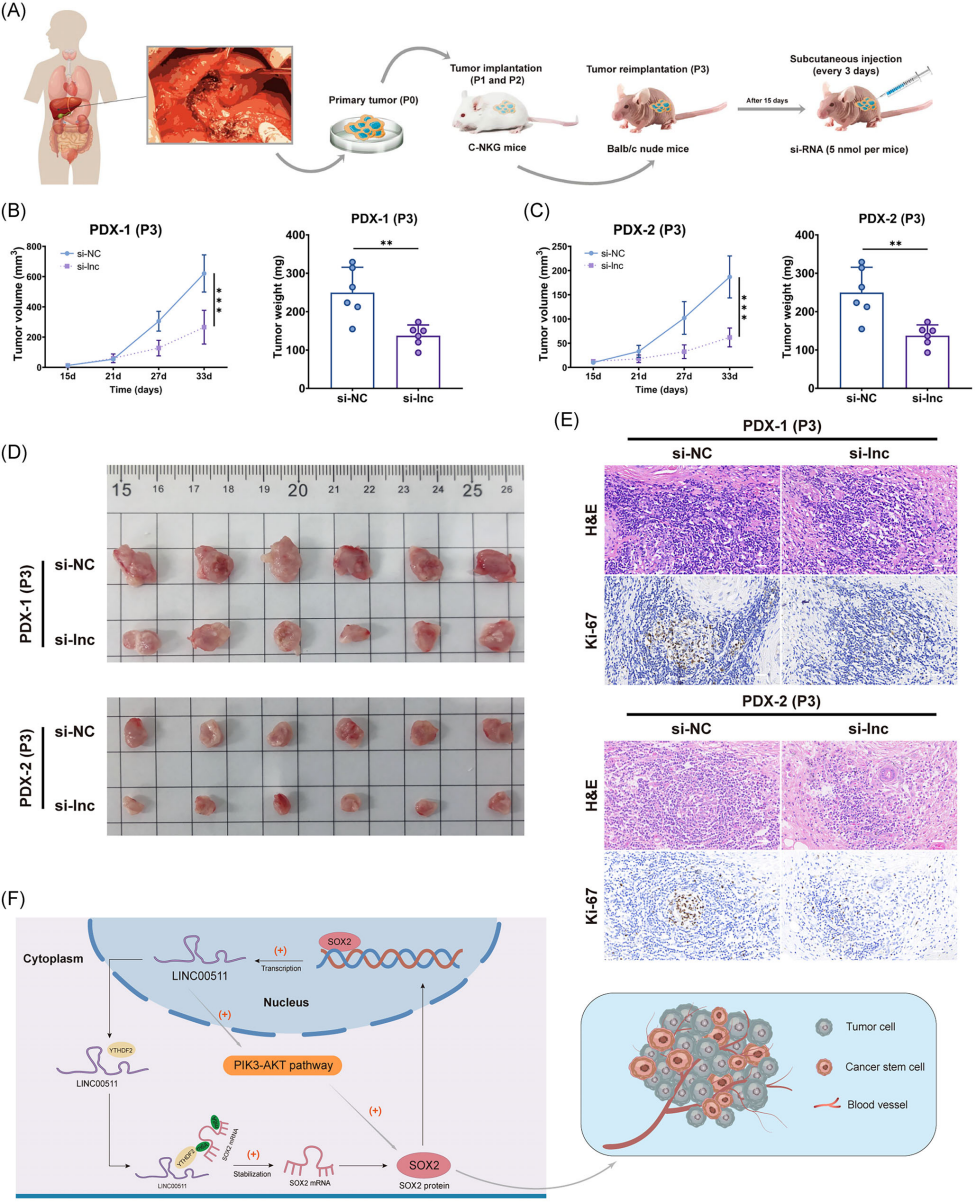
Patient‐derived xenografts (PDX) model results after intervention LINC00511. (A) The schematic diagram of PDX model construction. (B) Tumor weight and growth curves of the PDX‐1 post‐si‐LINC00511 injection (*n *= 6). (C) Tumor weight and growth curves of the PDX‐2 (*n *= 6). (D) Images of subcutaneous tumors in PDX models after using si‐LINC00511. (E) Hematoxylin and eosin staining and Ki‐67 immunohistochemistry of the PDX model tissues. (F) Regulatory mechanism schematic diagram of the LINC00511–YTHDF2–SOX2 positive feedback loop in cholangiocarcinoma. **p < 0.01, ***p < 0.001.

Two patient‐derived xenografts (PDX) models, named PDX‐1 and PDX‐2, were successfully established using NKG mice to mimic the growth environment of human cholangiocarcinoma (Figure 7A). This setup enabled the in vivo investigation of CCA in a controlled setting. Following the visible proliferation of the subcutaneous tumor, cholesterol‐modified siRNA targeting LINC00511 was administered to the si‐lnc group every three days for 18 days. The results showed a marked reduction in subcutaneous tumor growth in the PDX model of the si‐lnc group, indicating that targeted intervention with LINC00511 was effective in vivo (Figure 7B–D). Additionally, examination of tissue sections from PDX tissues showed that Ki‐67 expression was substantially reduced in the si‐lnc group (Figure 7E). Overall, the suppression of LINC00511 curtailed tumor growth and altered the expression of related biomarkers, providing crucial insights for understanding the molecular mechanism of CCA and advancing the development of new therapeutic strategies.

## 3 DISCUSSION

LncRNAs are RNA molecules exceeding 200 nucleotides long that they encode almost no proteins.^37^, ^38^ Recent research has highlighted the critical role of lncRNA in gene regulation, including transcriptional regulation, chromatin remodeling, and the regulation of transposon elements.^15^, ^21^, ^39^ Furthermore, lncRNAs were involved in various biological processes such as cell proliferation, migration, and apoptosis, making their role in tumor development a topic of widespread interest.^40^, ^41^ Cholangiocarcinoma was one of the most prevalent and deadly tumors of the digestive system globally, with an increasing incidence negatively impacting patient health and survival rates.^42^, ^43^, ^44^ Elucidating the pathogenesis of CCA, particularly the role of lncRNA, holds significant clinical implications.^45^ LINC00511 was a lncRNA known to play vital roles in various cancers.^46^, ^47^, ^48^ However, our understanding of the role and mechanism of LINC00511 in CCA remained limited. Thus, our study sought to investigate the expression, potential biological functions, and mechanisms of LINC00511 in cholangiocarcinoma.

Our research demonstrated that LINC00511 was significantly upregulated in CCA, aligning with other studies that had found increased expression levels of LINC00511 in malignancies such as non‐small‐cell lung cancer, liver cancer, glioma, and breast cancer.^49^, ^50^, ^51^ Furthermore, we discovered that high expression of LINC00511 correlated with adverse clinical characteristics and a poor survival prognosis, underscoring the significant role of LINC00511 in cancer onset and progression.

We also discovered that LINC00511 influence the biological functions of CCA tumor cells, including cell proliferation, migration, invasion, and tumorigenicity. These findings align with other tumor research, which suggests that LINC00511 could affect the biological functions of CCA by impacting the cell cycle and apoptosis.^52^, ^53^ Moreover, LINC00511 had been shown in previous studies to promote cancer through various mechanisms, such as competitively binding with microRNAs to downstream oncogenic mRNAs, activating multiple oncogenic signaling pathways, and recruiting proteins to regulate downstream gene expression.^54^, ^55^ Our investigation further elucidated that LINC00511 formed a complex with YTHDF2 and SOX2, significantly impacting SOX2 expression. This interaction was critical as it stabilized SOX2 mRNA through m6A methylation facilitated by YTHDF2. Additionally, YTHDF2 knockdown in normal bile duct cells did not affect SOX2 mRNA levels, suggesting a CCA‐specific dependency on YTHDF2 for SOX2 mRNA stability. This discovery provided a fresh perspective on the mechanistic role of LINC00511 in carcinoma. Additionally, our study found that SOX2, acting as a transcription factor, can promote the expression of LINC00511, thereby further influencing the biological functions of CCA cells. This discovery revealed a novel regulatory mechanism, the LINC00511–YTHDF2–SOX2–LINC00511 positive feedback loop, that plays a critical role in the onset and progression of cholangiocarcinoma (Figure 7F).

Our study also explored the potential clinical applications of LINC00511. The findings indicated that LINC00511 expression levels could serve as a potential biomarker, predicting clinical characteristics and survival prognosis. Additionally, through the construction of a CCA patient‐derived xenograft model, we found that targeted intervention with LINC00511 was effective in vivo, prompting the consideration of new therapeutic strategies targeting LINC00511 to improve the prognosis and survival rates of patients.

In summary, this research provides a comprehensive understanding of the role of LINC00511 in the pathogenesis of CCA. Our findings highlight the significance of the LINC00511–YTHDF2–SOX2 regulatory network in driving CCA progression and maintaining cancer stemness. The identification of m6A methylation as a key mechanism in stabilizing SOX2 mRNA underscores the importance of posttranscriptional modifications in cancer biology. This study not only elucidates the complex interplay between LINC00511, YTHDF2, and SOX2 but also proposes potential therapeutic targets within this regulatory axis for cholangiocarcinoma treatment.

## 4 MATERIALS AND METHODS

### 4.1 Clinical samples

This study collected 79 pairs of tumor tissues and adjacent tissues from cholangiocarcinoma patients at The 2nd Affiliated Hospital of Harbin Medical University. These patients underwent surgical treatment between March 2012 and May 2017 and had not received radiation or chemotherapy prior to surgery. All samples were immediately preserved in liquid nitrogen postsurgery until further experimental analysis. All patients provided informed consent, and the Ethics Committee of The 2nd Affiliated Hospital of Harbin Medical University approved this study (KY2022‐046). The clinical pathological data collected included age, sex, serum CEA levels, HBV infection status, and other relevant information.

### 4.2 Cell culture and transfection

Human umbilical vein endothelial cells (HUVEC) and HEK‐293T were acquired from the Cell Bank of the Chinese Academy of Sciences. Additionally, the normal bile duct cell line HIBEC and five cholangiocarcinoma cell lines, including CCLP‐1, QBC939, RBE, HuCCT1, KMBC, and TFK‐1, all of which were maintained in our laboratory. All cells were cultured in DMEM (Gibco) supplemented with 10% FBS (Invitrogen) at 37°C and 5% CO_2_. For transfection experiments, CCLP‐1, which exhibited the highest expression of LINC00511, was selected for knockdown experiments. Conversely, QBC939, with the lowest LINC00511 expression, was chosen for overexpression experiments. The lipofectamine 2000 transfection reagent (Invitrogen) was selected for transfections following the manufacturer's instructions. For LINC00511 knockdown, siRNA/shRNA targeting LINC00511 and control siRNA/shRNA (GenePharma) were utilized. For LINC00511 overexpression, a plasmid containing the sequence of LINC00511 and a control plasmid (GenePharma) were employed. The sequences involved are shown in Table S3.

### 4.3 qRT‐PCR

Total RNA was extracted from clinical and cell samples using TRIzol reagent (Invitrogen) according to the manufacturer's instructions. Then, 1 µg of total RNA was reverse transcribed into cDNA using the Transcriptor First Strand cDNA Synthesis Kit (Roche). Subsequently, FastStart Universal SYBR Green Master (Roche) was used to conduct qRT‐PCR. GAPDH was used as an internal control gene, and relative gene expression levels were calculated using the 2^−ΔΔCT^ method. The primer sequences are shown in Table S1.

### 4.4 Cell viability and apoptosis assays

CCK‐8 and EdU assays were utilized to assess cellular proliferative capacity. For the CCK‐8 assay, transfected cells were seeded in a 96‐well plate with each well containing 1 × 10^4^ cells. At specified time points (24, 48, 72, and 96 h), 10 µL of CCK‐8 reagent (Dojindo) was added to each well, followed by incubation at 37°C for 2 h. Then, the absorbance was measured at 450 nm using a microplate reader (Tecan). For the EdU assay, the EdU Apollo567 in vitro imaging kit (Ribobio) was used following the manufacturer's instructions. Specifically, transfected cells were seeded in a 96‐well plate with each well containing 1 × 10^4^ cells. At predetermined time points, 50 µM EdU labeling reagent was added to each well, followed by incubation at 37°C for 2 h. Subsequently, the cells were fixed, stained, and imaged using a fluorescence microscope (Leica). The ratio of EdU‐positive (red) and Hoechst‐positive (blue) cells was calculated to evaluate cell proliferation.

According to the manufacturer's instructions, the TUNEL assay was used to detect cell apoptosis using the In Situ Cell Death Detection Kit (Roche). Transfected cells were seeded onto glass slides and then fixed and permeabilized. The TUNEL reaction mixture was added to each sample and incubated at 37°C for 1 h. Then, imaging was performed using a fluorescence microscope. The ratio of TUNEL‐positive (green) and Hoechst‐positive (blue) cells was calculated to assess the cell apoptosis rate.

### 4.5 Metastasis assays

For the wound‐healing assay, after transfection, cells were seeded in a six‐well plate, each well containing 5 × 10^5^ cells, and cells were allowed to grow to 90−100% confluence. A straight line was scratched in each well using a 200 µL pipette tip. Then, images were captured at 0 and 48 h using an inverted microscope (Leica), and the width of the scratch was measured using ImageJ software (NIH).

Transwell assays were employed to assess the migration and invasion capabilities of cells. For the migration assay, transfected cells (for instance, 5 × 10^4^) were suspended in a medium containing 1% fetal bovine serum, and the cells were added to the upper chamber of the transwell insert (Corning). Medium containing 10% FBS was added to the lower chamber as a chemoattractant. After incubation at 37°C for 24 h, the nonmigrated cells in the upper chamber were removed with a cotton swab, and then the migrated cells in the lower chamber were fixed and stained. Images were captured using an inverted microscope, and the number of migrated cells was calculated. The invasion assay was identical to the migration assay, but Matrigel (BD) was precoated in the upper chamber before adding cells. After incubation at 37°C for 48 h, the cells that had invaded the lower chamber were fixed, and stained, images were captured, and the cells were counted.

### 4.6 Tumor sphere formation assay

The tumor sphere formation assay was employed to evaluate the tumorigenic stemness of cells. In essence, transfected cells were suspended in a serum‐free medium containing B27, EGF, and bFGF (MedChemExpress) and seeded at a density of 1 × 10^3^ cells/well in an ultralow attachment six‐well plate (Corning). The cells were incubated at 37°C for 7−14 days, allowing for tumor sphere formation. Then, images were captured using a microscope, and the number and size of the formed tumor spheres were calculated using ImageJ software. Tumor spheres with diameters larger than 50 µm were counted as valid spheres. All experiments were repeated at least three times.

### 4.7 Angiogenesis assay

This assay evaluated the angiogenic capacity of cells. Specifically, conditioned media were collected from transfected cells. Then, HUVEC was suspended in a medium containing 2% fetal bovine serum, and the cells were added to a 24‐well plate precoated with Matrigel. The cells were incubated at 37°C for 6−8 h, allowing for tubular structure formation. Subsequently, images were captured using an inverted microscope, and the number and length of the formed tubular structures were measured using ImageJ software.

### 4.8 Western blot

Initially, cells were collected after transfection, total protein was extracted using RIPA buffer (Beyotime), and the protein concentration was determined using a BCA protein assay kit (Beyotime). Subsequently, electrophoresis of protein samples was performed on SDS‐PAGE gels, and the proteins were transferred onto PVDF membranes (Millipore). The membrane was blocked with 5% nonfat milk and was incubated overnight at 4°C with specific primary antibodies. Following this, the membranes were incubated with corresponding secondary antibodies for 1 h at room temperature, and signals were detected using an ECL detection system (Beyotime). ImageJ software quantified the protein band intensity and normalized it to GAPDH as an internal reference.

### 4.9 Subcellular localization assay

In essence, cells were collected posttransfection and separated into cytoplasmic and nuclear fractions following the manufacturer's instructions using a subcellular fractionation kit (Life Technologies). Then, RNA was extracted from each fraction, and LINC00511 expression was detected using qRT‐PCR. GAPDH was used as a cytoplasmic internal reference and U6 as a nuclear internal reference. Finally, the relative expression of LINC00511 was calculated in the cytoplasmic and nuclear fractions, thus determining its principal location within the cell. All experiments were performed in triplicate.

### 4.10 Fluorescence in situ hybridization

FISH was used to validate the localization of LINC00511 and SOX2 within the cell. Simplistically, the posttransfected cells were seeded onto glass slides and fixed using 4% formaldehyde. Cells were treated with 0.5% Triton X‐100 to enhance cell membrane permeability. Afterward, cells were pretreated with a hybridization solution (Ribobio) and incubated overnight at 37°C in a hybridization solution containing the LINC00511 or SOX2 probe (Ribobio). Images were then captured using a fluorescence microscope.

### 4.11 RNA pull‐down

Following the manufacturer's instructions (Promega), LINC00511 was initially transcribed into biotin‐labeled RNA and incubated with cell lysate to allow RNA–protein interactions. Then, magnetic beads were added to bind with biotin and incubated overnight at 4°C to capture biotin‐labeled RNA and associated proteins. Magnetic separation was used to isolate the beads, and they were washed to remove nonspecifically bound proteins. Subsequently, SDS‐PAGE electrophoresis was performed, and protein expression was detected using western blot.

### 4.12 RNA immunoprecipitation

The RNA immunoprecipitation (RIP) experiment validated the interaction among LINC00511, YTH N6‐methyladenosine RNA binding protein F2 (YTHDF2), and SRY‐box transcription factor 2 (SOX2). Total protein was extracted using IP buffer, and then protein samples were incubated with specific antibodies (such as anti‐YTHDF2) overnight at 4°C to form immune complexes. Protein A/G magnetic beads (Invitrogen) were added to capture the immune complexes and incubated overnight at 4°C. Magnetic separation was used to isolate the beads, and they were washed to remove nonspecifically bound proteins. Subsequently, LINC00511 and SOX2 expression were detected using qRT‐PCR.

### 4.13 Immunofluorescence

The cellular immunofluorescence experiment was employed to observe the localization of YTHDF2 within the cell. In essence, posttransfection cells were initially seeded onto glass slides and fixed using 4% formaldehyde. Then, the cells were treated with 0.1% Triton X‐100 to enhance cell membrane permeability and 5% BSA to block nonspecific binding sites. The cells were incubated overnight at 4°C with primary antibodies and incubated with fluorescently labeled secondary antibodies for 1 h at room temperature. The cell nuclei were stained with DAPI, and pictures were taken using a fluorescence microscope.

### 4.14 Actinomycin D assay

Actinomycin D (Sigma) was utilized to observe the influence of LINC00511 and YTHDF2 on SOX2 mRNA stability. In essence, posttransfection cells were initially treated with a medium containing 5 µg/mL actinomycin D to inhibit new mRNA synthesis. Then, cells were collected at specific time points (0, 4, 8, and 12 h), and total RNA was extracted. qRT‐PCR was used to detect SOX2 mRNA expression at different time points.

### 4.15 Methylated RNA immunoprecipitation

The purpose of the MeRIP experiment was to assess the methylation status of SOX2 mRNA. Total RNA was extracted from cholangiocarcinoma cells using TRIzol reagent (Invitrogen), followed by DNase I treatment (Roche) to remove any DNA contamination.

Using a Magna MeRIP m6A Kit (17‐10499) following the manufacturer's recommendations. To enrich m6A‐modified mRNA, DNA‐free fragmented RNAs were incubated with an anti‐m6A antibody in RIP buffer at 4°C for 2 h. This step was followed by the addition of prewashed protein A/G magnetic beads (Invitrogen) and further incubation for 2 h at 4°C to capture the antibody–RNA complexes. After incubation, the beads were washed with RIP buffer to remove nonspecific interactions. The eluted RNA was then purified using phenol and ethanol precipitation and was analyzed using quantitative PCR to quantify the enrichment of m6A‐modified SOX2 mRNA compared with the input control.

### 4.16 Chromatin immunoprecipitation

Following the ChIP instructions provided by Millipore, the cells were initially fixed using 1% formaldehyde to form protein–DNA crosslinks. Subsequently, the chromatin was sonicated into 200−500 bp fragments and incubated overnight at 4°C with specific primary antibodies (rabbit IgG as a negative control). Protein A/G magnetic beads were added to capture the antibody–chromatin complexes, followed by overnight incubation at 4°C. The magnetic beads were separated using a magnet and washed to remove nonspecifically bound DNA, and the protein was then digested with proteinase K. DNA was extracted using phenol‐chloroform. qRT‐PCR was used to detect the DNA of the LINC00511 promoter region and calculate its relative enrichment in the immunoprecipitation sample.

### 4.17 Luciferase reporter gene assay

Luciferase reporter gene assay was employed to validate whether SOX2 could regulate the promoter activity of LINC00511. Basically, the promoter region of LINC00511 was cloned and inserted into the luciferase reporter gene vector pGL3 (Promega) to create the reporter gene plasmid. Subsequently, the reporter gene plasmid and the plasmid overexpressing SOX2 or an empty vector were co‐transfected into cells. After 48 h, luciferase activities were measured using a luciferase reporter gene detection system (Promega). All experiments were performed in triplicate.

### 4.18 In vivo assay

Transfected tumor cells were suspended in PBS and subcutaneously injected into the dorsal side of nude mice (Cyagen Biosciences) to establish a subcutaneous tumor model. The length and width of the tumor were intermittently measured, and its volume was calculated. A few weeks later, the mice were euthanized, and the tumors were excised and weighed. Immunohistochemistry (IHC) was employed to examine Ki‐67 expression in the tumor and assess cell proliferation.

For the hepatic metastasis model, transfected CCA cells were suspended in PBS and injected subcapsular into the spleen of nude mice. Weeks later, in vivo imaging system (Thermo Fisher Scientific) was utilized to detect hepatic metastasis within the mice. Then, the mice were euthanized, and the liver was excised to observe the quantity and size of the metastatic tumors. H&E staining was used to examine the metastatic tumors in liver sectiosns.

To construct PDX models, fresh tumor tissue samples were initially procured from CCA patients. These samples were immediately processed under sterile conditions post‐procurement, including washing, cutting, and dividing. These cut tumor tissues (P0) were transplanted into c‐NKG mice (Cyagen Biosciences) for propagation and expansion. After successful transplantation and propagation through two generations (P1 and P2), a sufficient amount of tumor tissue was obtained for subsequent experiments. Then, these second‐generation (P2) tumor tissues were transplanted subcutaneously into nude mice to construct the PDX model. After successfully constructing two independent PDX models, they were denominated PDX‐1 and PDX‐2. Fourteen days after successful tumor transplantation and the commencement of growth, intervention treatments were initiated in the PDX model. Specifically, cholesterol‐modified siRNA (Ribobio) targeting LINC00511 was subcutaneously injected every three days. After 15 days of treatment, the mice were euthanized, and the tumor volume and weight were measured. The Ethics Committee of The 2nd Affiliated Hospital of Harbin Medical University approved all animal experiments (SYDW2022‐036).

### 4.19 IHC and H&E assays

Paraffin IHC and H&E assays were used to observe cell changes in tumor tissues and liver sections. Briefly, the tumor and liver sections were fixed in formalin and underwent a series of steps to produce paraffin sections, including dehydration, transparency, wax immersion, and embedding. For IHC, antigen retrieval was conducted in the sections using a microwave oven, followed by blocking endogenous peroxidase with 3% hydrogen peroxide. Subsequently, nonspecific binding was blocked with normal sheep serum, and the sections were incubated with the Ki‐67 antibody. Then, the sections were incubated with secondary antibodies, visualized with DAB (Beyotime), and counterstained with H&E (Beyotime). Finally, the sections were observed under a microscope, and the proportion of Ki‐67‐positive cells was calculated.

### 4.20 Statistical analysis

All statistical analyses were conducted using SPSS software (IBM SPSS). For normally distributed data, independent sample *t*‐tests or one‐way analysis of variance were used for comparisons. For nonnormally distributed data, Mann–Whitney *U*‐ and Kruskal–Wallis tests were utilized. Pearson correlation analysis was used to study the relationships between two continuous variables. For categorical variables, the chi‐square test or Fisher's exact test was used for comparison. Finally, the Kaplan–Meier and log‐rank tests were used to analyze survival rates, and the Cox proportional hazards model was used to analyze factors affecting survival. *P*‐value less than 0.05 was considered statistically significant.

## AUTHOR CONTRIBUTIONS

Canghai Guan, Xinlei Zou, and Xin Gao designed and executed the experiments. Xinlei Zou and Sidi Liu collected the data. Xingming Jiang and Xiangyu Zhong provided the experimental resources. Jianjun Gao, Wujiang Shi, and Qingfu Dong wrote and modified the manuscript. Xingming Jiang and Xiangyu Zhong supervised the manuscript. All authors have read and approved the final manuscript.

## CONFLICT OF INTEREST STATEMENT

The authors declare no conflict of interest.

## ETHICS STATEMENT

Human tissue usage was sanctioned by The 2nd Affiliated Hospital of Harbin Medical University. The execution of all procedures strictly adhered to the approved guidelines (SYDW2022‐036). All animal studies strictly adhered to approved protocols by The 2nd Affiliated Hospital of Harbin Medical University (KY2022‐046).

## Supporting information



Supporting Information

## Data Availability

The datasets used and/or analyzed during the current study are derived from the publicly accessible TCGA (The Cancer Genome Atlas, https://portal.gdc.cancer.gov/projects/TCGA‐CHOL) database. Other experimental data and additional information are available from the corresponding author upon reasonable request.
