# Supplementary material for: Feedback loop LINC00511–YTHDF2–SOX2 regulatory network drives cholangiocarcinoma progression and stemness
Source: MedComm (2020). 2024 Oct 22;5(11):e743. doi: 10.1002/mco2.743 (PMC11496568; doi:10.1002/mco2.743)
Supplement: Supplementary file 1 — Supporting Information [file MCO2-5-e743-s001.doc]

***Supplementary Material***

**Title**

Feedback loop LINC00511-YTHDF2-SOX2 regulatory network drives cholangiocarcinoma progression and stemness

**Running title**

LINC00511 in cholangiocarcinoma

Canghai Guan^1,#^, Xinlei Zou^1,#^, Xin Gao^1,#^, Sidi Liu^1,#^, Jianjun Gao^1^, Wujiang Shi^1^, Qingfu Dong^1^, Xingming Jiang^1,*^, Xiangyu Zhong^1,*^

^1^General Surgery Department, The 2nd Affiliated Hospital of Harbin Medical University, 148 Baojian Street, Harbin 150086, Heilongjiang Province, China.

^#^These authors contributed equally: Canghai Guan, Xinlei Zou, Xin Gao, Sidi Liu.

^*^Corresponding author: **Xingming Jiang**, General Surgery Department, The 2nd Affiliated Hospital of Harbin Medical University, 148 Baojian Street, Harbin 150086, Heilongjiang Province, China. E-mail: xmjiang@hrbmu.edu.cn; and **Xiangyu Zhong**, General Surgery Department, The 2nd Affiliated Hospital of Harbin Medical University, 148 Baojian Street, Harbin 150086, Heilongjiang Province, China. E-mail: hmuzhongxy@163.com.

**Supplementary Table 1** Primer sequences for qRT-PCR and siRNAs sequences

| Nucleic acids | Sequences |
| --- | --- |
| GAPDH | F: 5’-GAAGGTGAAGGTCGGAGT-3’ |
|  | R: 5’-GAAGATGGTGATGGGATTTC-3’ |
| U6 | F: 5’-GCTTCGGCAGCACATATACTAAA-3’ |
|  | R: 5’-CGCTTCACGAATTTGCGTGTC-3’ |
| LINC00511 | F: 5’-AGGGGCGACTACTGTTACCT-3’ |
|  | R: 5’-CGTCCAAACAGGCTGGATCT-3’ |
| E1 | F: 5’-GAGGCTCTTTGAGGACTGGG-3’ |
|  | R: 5’-GAGGCTCTTTGAGGACTGGG-3’ |
| E2 | F: 5’-TCAGCTGGTAACTTCGGGAC-3’ |
|  | R: 5’-TCCTGTTAAGCCATGCCACA-3’ |
| E3 | F: 5’-GGGCCTCATATGCTTGACTGT -3’ |
|  | R: 5’-TCACCTGGAAGAAGGGTCCA-3’ |
| SOX2 | F: 5’-GCCCTGCAGTACAACTCCAT-3’ |
|  | R: 5’-GACTTGACCACCGAACCCAT-3’ |
| YTHDF2 | F: 5’-CAGTCTCAGGCTGGTTCTGG-3’ |
|  | R: 5’-CTCTCTGGTTCCTCCTTCCCTA-3’ |
| si-NC | 5’-TTCTCCGAACGTGTCA-3’ |
| si-lnc-1 | 5’-GGTGAGTCTTTGTCCTTAAAG-3’ |
| si-lnc-2 | 5’-GGATGTGTCTGTTACAATTCA-3’ |
| si-lnc-3 | 5’-GGTGTGTTAAATTAGTAAATT-3’ |
| si-NC | 5’-TTCTCCGAACGTGTCACGTTT-3’ |
| si-YTHDF2 | 5’-GCTGGATGATCAAGATCTAAG-3’ |
| si-SOX2 | 5’-CAGTATTTATCGAGATAAACA-3’ |

**Supplementary Table 2 Correlation between LINC00511 expre**ssion and clinicopathological characteristics of CCA patients.

| **Clinicopathological parameters** | **Total Cases**  **(n=79)** | **LINC00511 expression** | | ***P*** **value** |
| --- | --- | --- | --- | --- |
|  |  | **Low (n=40)** | **High (n=39)** |  |
| Age (years) |  |  |  | 0.4355 |
| <60 | 33 | 15 | 18 |  |
| ≥60 | 46 | 25 | 21 |  |
| Gender |  |  |  | 0.4173 |
| Male | 34 | 19 | 15 |  |
| Female | 45 | 21 | 24 |  |
| Smoking |  |  |  | 0.2176 |
| No | 42 | 24 | 18 |  |
| Yes | 37 | 16 | 21 |  |
| Alcoholic |  |  |  | 0.4342 |
| No | 37 | 17 | 20 |  |
| Yes | 42 | 23 | 19 |  |
| TNM stage |  |  |  | 0.0321 |
| I-II | 38 | 24 | 14 |  |
| III-IV | 41 | 16 | 25 |  |
| Lymph node invasion |  |  |  | 0.0087 |
| Positive | 31 | 10 | 21 |  |
| Negative | 48 | 30 | 18 |  |
| Vascular invasion |  |  |  | 0.0709 |
| Positive | 23 | 8 | 15 |  |
| Negative | 56 | 32 | 24 |  |
| HBV infection |  |  |  | 0.5681 |
| Positive | 37 | 20 | 17 |  |
| Negative | 42 | 20 | 22 |  |
| Serum CEA (ng/ml) |  |  |  | 0.4173 |
| >5 | 45 | 21 | 24 |  |
| ≤5 | 34 | 19 | 15 |  |
| Serum CA19-9 (U/ml) |  |  |  | 0.2139 |
| >37 | 41 | 18 | 23 |  |
| ≤37 | 38 | 22 | 16 |  |

*Bold values indicate P< 0.05*

Supplementary Table 3 Univariate and multivariate analysis for overall survival of CCA patients.

| **Variables** | **Univariate analysis** | | | **Multivariate analysis** | | |
| --- | --- | --- | --- | --- | --- | --- |
|  | **HR** | **95% CI** | ***P* value** | **HR** | **95% CI** | ***P* value** |
| Age (years) |  |  |  |  |  |  |
| ≥60 *vs*. <60 | 0.669 | 0.356-1.257 | 0.212 |  |  |  |
| Gender |  |  |  |  |  |  |
| Male *vs*. Female | 0.932 | 0.493-1.760 | 0.827 |  |  |  |
| Vascular invasion |  |  |  |  |  |  |
| Positive *vs*. Negative | 1.682 | 0.876-3.233 | 0.119 |  |  |  |
| HBV infection |  |  |  |  |  |  |
| Positive *vs*. Negative | 0.781 | 0.413-1.479 | 0.449 |  |  |  |
| Serum CEA (ng/ml) |  |  |  |  |  |  |
| >5 *vs*. ≤5 | 1.143 | 0.607-2.152 | 0.679 |  |  |  |
| Serum CA19-9 level (U/ml) |  |  |  |  |  |  |
| >37 *vs*. ≤37 | 0.769 | 0.410-1.445 | 0.415 |  |  |  |
| Lymph node invasion |  |  |  |  |  |  |
| Positive *vs*. Negative | 2.369 | 1.251-4.484 | 0.008** | 2.261 | 1.120-4.564 | **0.023** |
| TNM stage |  |  |  |  |  |  |
| III-IV *vs*. I-II | 2.363 | 1.175-4.754 | 0.016* | 1.791 | 0.838-3.825 | 0.133 |
| LINC00511 expression |  |  |  |  |  |  |
| Low *vs*. High | 2.491 | 1.300-4.774 | 0.006** | 3.155 | 1.561-6.377 | **0.001** |

*Bold values indicate P< 0.05*


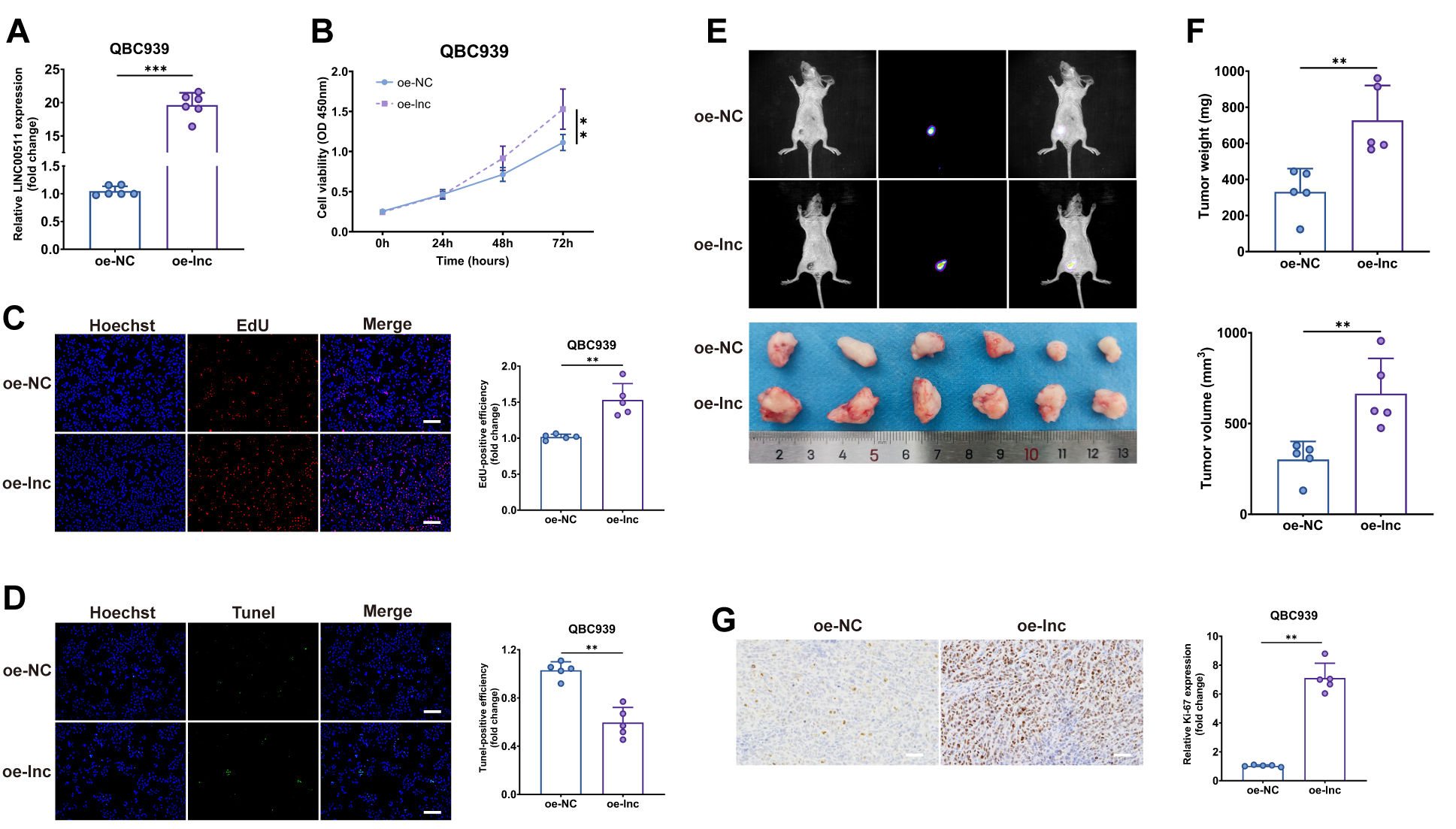


**Fig. S1 Impact of LINC00511 on CCA proliferation and apoptosis.** **A** Intervention efficiency after transfection with an overexpression vector (*n*=6). **B, C** Assessment of proliferation alterations in QBC939 through CCK-8 and EdU assays (*n*=6). **D** Apoptosis alterations of QBC939 evaluated via Tunel assays (*n*=6). **E** Representative images of subcutaneous tumors in nude mice after LINC00511 overexpression. **F** Weight and volume of subcutaneous tumors post LINC00511 overexpression (*n*=5). **G** Subcutaneous tumor tissues from different groups were subjected to Ki-67 staining (*n*=5). ***P*<0.01, ****P*<0.001.


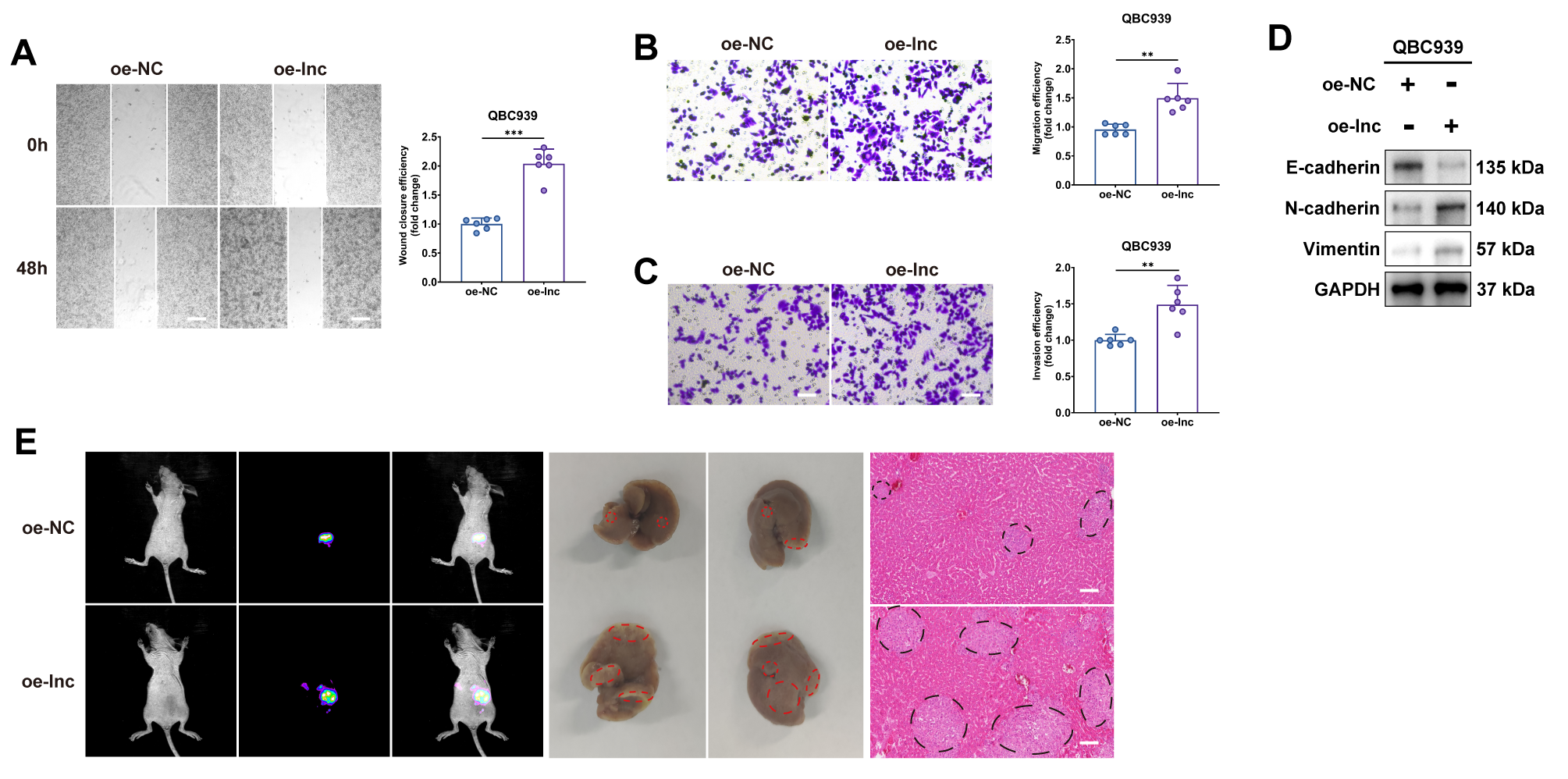


**Fig. S2 Influence of LINC00511 on CCA migration and invasion.** **A, B** Wound healing and transwell migration assays gauging the migratory capability of QBC939 (*n*=6). **C** Transwell invasion assays determining the invasive potential of QBC939 (*n*=6). **D** Western blot analysis of EMT-related markers. **E** Representative images of liver metastasis following overexpression of LINC00511 ***P*<0.01, ****P*<0.001.


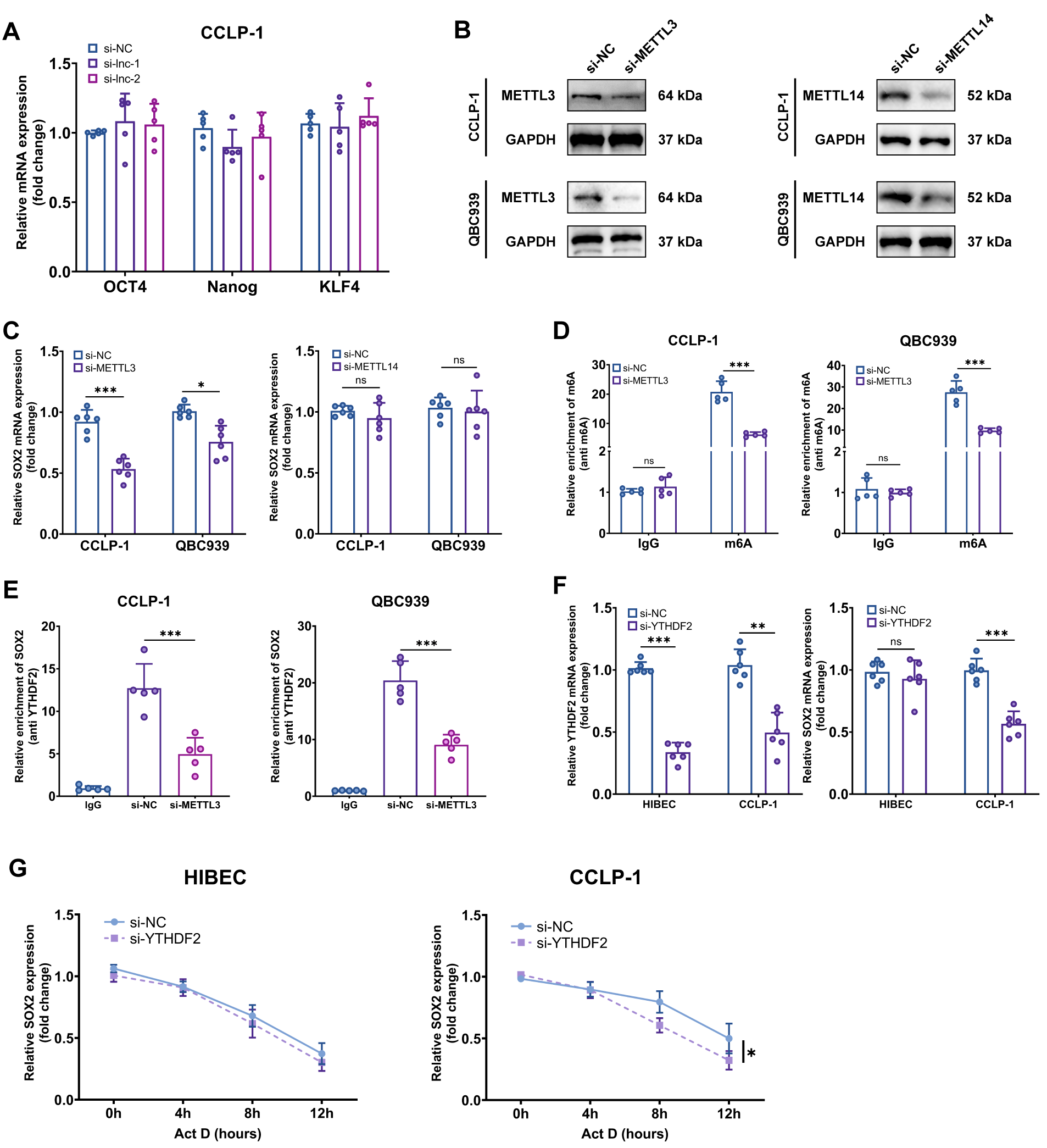


**Fig. S3 YTHDF2 stabilized SOX2 mRNA in CCA in an m6A-dependent manner.** **A** Expression levels of OCT4, Nanog, and KLF4 after silencing LINC00511 (*n*=6). **B** Western blot analysis showing the knockdown efficiency of METTL3 and METTL14 (*n*=6). **C** Expression levels of SOX2 mRNA after silencing METTL3 and METTL14 in CCA cells (*n*=6). **D** MeRIP-qPCR analysis of the m6A levels of SOX2 mRNA in CCA cells following METTL3 knockdown (*n*=6). **E** Enrichment of SOX2 mRNA in the RIP of YTHDF2 in CCLP-1 and QBC939 cells after METTL3 knockdown (*n*=6). **F** SOX2 mRNA expression in HIBEC and CCLP-1 cells after METTL3 knockdown (*n*=6). **G** Evaluation of SOX2 mRNA stability over time in HIBEC and CCLP-1 cells transduced with si-YTHDF2 or si-NC, following actinomycin D treatment (*n*=6). **P*<0.05, ***P*<0.01, ****P*<0.001.
